# Decoding Treatment Failures in Metastatic Renal Cell Carcinoma: Predictors Across Immunotherapy and Targeted Therapies from a Retrospective Real-World Analysis

**DOI:** 10.3390/jcm14155271

**Published:** 2025-07-25

**Authors:** Sorin Saftescu, Vlad-Norin Vornicu, Dorel-Ionel Popovici, Radu-Dumitru Dragomir, Dana-Sonia Nagy, Daniela-Lidia Sandu, Ana Dulan, Șerban-Mircea Negru, Alina-Gabriela Negru

**Affiliations:** 1 Department of Oncology, Faculty of Medicine, Victor Babes University of Medicine and Pharmacy Timisoara, Eftimie Murgu Square 2, 300041 Timisoara, Romania; sorin.saftescu@umft.ro (S.S.); vlad.vornicu@umft.ro (V.-N.V.); radu.dragomir@umft.ro (R.-D.D.); dana.nagy@umft.ro (D.-S.N.); daniela.sandu@umft.ro (D.-L.S.); ana.dulan@umft.ro (A.D.); serban.negru@umft.ro (Ș.-M.N.); 2 Department of Cardiology, Faculty of Medicine, Victor Babes University of Medicine and Pharmacy Timisoara, Eftimie Murgu Square 2, 300041 Timisoara, Romania; alinanegru@umft.ro

**Keywords:** metastatic renal cell carcinoma, time to treatment failure, real-world evidence, predictors, immunotherapy, targeted therapy

## Abstract

**Background**: Despite recent advances in the management of metastatic renal cell carcinoma (mRCC), real-world outcomes remain heterogeneous, and early treatment failure is common. Predictive biomarkers for time to treatment failure (TTF) outside clinical trials are poorly characterized. **Objective**: To identify clinical and laboratory predictors associated with early treatment failure in a real-world cohort of mRCC patients treated with immune checkpoint inhibitors (ICIs), tyrosine kinase inhibitors (TKIs), or combination regimens. **Methods**: We conducted a retrospective, single-center analysis of patients with metastatic non-urothelial RCC treated between 2018 and 2023. Cox proportional hazards regression was used to evaluate the association between baseline biological parameters and TTF for each treatment regimen. **Results**: Among 137 patients receiving first-line therapy, 50 received Ipilimumab + Nivolumab, 49 Sunitinib, and 17 Avelumab + Axitinib. For Ipilimumab + Nivolumab, elevated AST was significantly associated with shorter TTF. For Avelumab + Axitinib, shorter TTF was associated with lymph node metastases, low lymphocyte count, low creatinine, low BMI, and low hemoglobin. For Cabozantinib in subsequent lines, a higher platelet count, ALT, and presence of liver metastases were associated with shorter TTF. No statistically significant predictors were found for Nivolumab used in the second-line setting. **Conclusions**: Routine, accessible biomarkers such as AST, hemoglobin, lymphocyte count, and creatinine may serve as predictors of treatment failure in specific therapeutic contexts. These findings support risk-adapted strategies and individualized monitoring in real-world clinical practice, though further validation in larger cohorts is warranted.

## 1. Introduction

According to the European Cancer Information System (ECIS), in 2022, the incidence of renal cell carcinoma (RCC) in the EU-27 was estimated at 58,228 new cases in males (26.8 per 100,000, age-standardized rate [ASR]) and 37,493 in females (12.2 per 100,000 ASR), totaling 95,721 cases (20.3 per 100,000 ASR for both sexes). Projections for 2040 estimate a 15.19% increase in annual cases. The highest incidence in the EU is reported in Czechia (29.4 per 100,000) and the lowest in Cyprus (8.7 per 100,000). Historical data show a consistent rise in incidence, from 13.2 per 100,000 in 1976 to 20.3 in 2022 [1]. Similar trends are reported globally, with an average annual increase of 2–3% in most countries [2]. This increasing incidence is attributed to improved detection and lifestyle factors such as the rising prevalence of obesity [3], hypertension, and smoking—though smoking rates in the EU have decreased from 30.5% in 2010 to 26.4% in 2019 [4].

Key risk factors for RCC include obesity (RR = 1.35 in overweight patients; RR = 1.76 for BMI (body mass index) ≥ 30 kg/m^2^), accounting for ~26% of RCC cases [5], smoking (RR = 1.38) [6], hypertension (RR = 1.05 per +10 mmHg systolic, RR = 1.07 per +10 mmHg diastolic) [7], male sex (RR = 2) [8], diabetes (RR = 1.4) [8], chronic kidney disease (RR = 1.39) [9], and height: (RR = 1.3 per + 10 cm) [10]. Conversely, kidney stones, sodium intake, and fluid intake do not have statistically significant associations with RCC risk [11,12], and alcohol consumption may be protective (RR = 0.71) [13].

While only 3–5% of RCC cases are familial—with von Hippel–Lindau (VHL) disease, an autosomal dominant inherited disorder, accounting for approximately 1% of cases [14]—about 50–60% of sporadic cases harbor biallelic VHL inactivation. This inactivation is caused by mutations, loss of heterozygosity at 3p, or promoter hypermethylation. Overall, 90% of sporadic ccRCCs demonstrate some form of VHL inactivation [15,16].

Clear cell renal cell carcinoma (ccRCC) accounts for approximately 65–70% of adult renal carcinomas. Less common subtypes include papillary RCC (10–15%) and chromophobe RCC (5%) [17,18].

**Figure 1:**
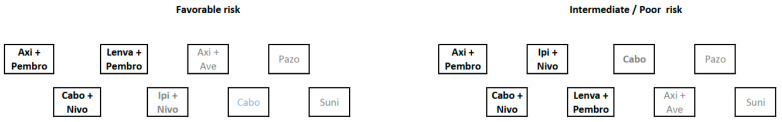
Axi = Axitinib, Pembro = Pembrolizumab, Cabo = Cabozantinib, Nivo = Nivolumab, Lenca = Lenvatinib, Ipi = Ipilimumab, Ave = Avelumab, Pazo = Pazopanib, Suni= Sunitinib. Preferred regimens in bold, category 1 in black, category 2A in gray, category 2B in blue.

The current therapeutic landscape (NCCN Guidelines, Version 3, 2025, regimens category 1, 2A or 2B listed) is presented in Figure 1 [19].

Table 1 outlines the key clinical trials that reflect the historical evolution of therapeutic strategies in renal cell carcinoma. 

When comparing IO–TKI to IO–IO combinations by efficacy trends, the progression-free survival (PFS) benefit of IO–TKI regimens appears greater. However, this advantage tends to diminish with longer follow-ups, and ongoing data maturation will clarify whether this trend significantly impacts long-term outcomes [28].

Real-world studies for mRCC indicate, for different cohorts (TKI, TKI+IO, IO–IO), a median duration of first-line treatment of 2.5–8.3 months and median survivals of between 39 and 42 months (Gu-Shun Lai et al., 2024), revealing a high proportion of cases with early treatment failure.

Real-world predictive factors identified included patients with bone or liver metastases, high C-reactive protein (CRP), paraneoplastic symptoms, or, without prior cytoreductive nephrectomy experience, shorter PFS on Ipilimumab + Nivolumab [29]. A large real-world analysis (*n* = 455) reported a median PFS of 13.5 months and a median OS of 51.5 months with Ipilimumab + Nivolumab. Predictors of response included male sex (OR 2.0, *p* = 0.017), prior nephrectomy (OR 2.1, *p* = 0.001), and lung metastasis (OR 2.4, *p* = 0.001). Better ECOG PS (0 or 1), IMDC intermediate risk, and completion of four induction cycles were also favorable, while antibiotic use before immunotherapy predicted a worse OS [30]. A smaller retrospective series (*n* = 48) suggested that normal or normalized CRP during treatment predicted longer PFS with Nivolumab + Ipilimumab [31]. High CRP (>1.27 mg/dL) also predicted shorter PFS in patients treated with Cabozantinib (*n* = 53), with a hazard ratio of 6.9 in univariate analysis, as was demonstrated in a real-world Japanese cohort study evaluating Cabozantinib outcomes (Iinuma et al., Biomedicines 2022, 10, 3172)

Over the past two decades, the management of metastatic renal cell carcinoma (mRCC) has evolved from a primary reliance on cytoreductive nephrectomy (CN) to a multimodal strategy increasingly centered on systemic therapies. Although observational and randomized studies have evaluated the role of CN in the era of targeted agents, optimal timing and patient selection remain matters of ongoing debate, highlighting the need for individualized, multidisciplinary treatment planning [32].

## 2. Materials and Methods

The objective of this study was to identify clinical and laboratory predictors associated with early treatment failure in patients with metastatic renal cell carcinoma (mRCC) treated with immune checkpoint inhibitors (ICIs), tyrosine kinase inhibitors (TKIs), or combination regimens in a real-world setting.

This retrospective single-center, observational cohort study was conducted at the OncoHelp Medical Center, Timișoara, Romania, a tertiary oncology referral center for patients with non-urothelial metastatic renal cell carcinoma (mRCC) treated in first and subsequent lines with ipilimumab + nivolumab, cabozantinib, sunitinib, pazopanib, nivolumab, or avelumab + axitinib at OncoHelp Hospital, Timisoara, Romania. The study fully complied with the ethical principles outlined in the Declaration of Helsinki, and ethical approval was obtained from the Ethics Committee of the OncoHelp Medical Center prior to study initiations (approval number 1178/2 May 2024).

Patients included in this retrospective study had histologically confirmed renal cell carcinoma with radiologically documented metastatic disease (M1), received at least one line of systemic therapy (ICI, TKI, or combinations) between February 2021 and July 2024, and had available baseline clinical and laboratory data; patients with non-metastatic disease, non-RCC histologies, or incomplete baseline records were excluded. No exclusion was applied based on comorbidities; the cohort included patients with various chronic conditions, such as cardiovascular, metabolic, or renal disorders.

Clinical and pathological data collected included age, sex, Eastern Cooperative Oncology Group (ECOG) performance status, histological subtype, prior treatment regimens, metastatic site locations, baseline complete blood count (CBC), and biochemistry at the start of each treatment line. We focused on routinely available clinical and laboratory parameters—such as AST, creatinine, lymphocyte count, and hemoglobin—given their widespread use in oncology practice, accessibility across care settings, and previous associations with prognosis or systemic treatment outcomes in solid tumors.

The diagnosis of renal cell carcinoma was confirmed in all patients through histopathological examination, based on the criteria outlined in the World Health Organization (WHO) Classification of Urinary and Male Genital Tumours, 5th edition (2022). All patients had radiological evidence of distant metastases (M1) documented prior to treatment initiation, confirmed by CT, MRI, or PET-CT according to institutional protocols.

The study analyzed 3576 treatment evaluations involving 137 unique patients diagnosed with ICD-10 C64 (renal cell carcinoma). For accurate treatment sequencing, 39 treatment lines initiated before the study period and 188 treatment lines initiated during the study period were identified and included in the analysis. Time to Treatment Failure (TTF) was defined as the time from the first to the last administration of a treatment line, with treatment discontinuation due to any cause (disease progression, toxicity, or death) considered as failure (Collett, D.—Modelling Survival Data in Medical Research, 3rd edition, CRC Press, 2015). The on-treatment status at the data cut-off was also recorded.

The primary objective was to identify predictive factors for prolonged time to treatment failure (TTF) for each therapeutic regimen. To achieve this, Cox proportional hazards models were constructed using Cox Proportional Hazards Survival Regression, based on baseline biological parameters obtained prior to the initiation of each treatment line.

No formal control group was included in this retrospective observational study. The analysis was descriptive and exploratory, focusing on identifying predictors of early treatment failure across different treatment cohorts. No blinding of participants or investigators was performed, as this was a retrospective observational study based on routinely collected real-world data.

No long-term clinical follow-up was conducted beyond treatment discontinuation, as the primary endpoint was time to treatment failure.

Cox proportional hazards regression analyses were performed using the online calculator available at https://statpages.info/prophaz.html, accessed on 22 March 2025, which implements the method described by Lee and Wang (2003). A two-tailed *p*-value of <0.05 was considered statistically significant. Given the small sample sizes in some subgroups and the potential interdependence between clinical and laboratory variables, we limited the analysis to univariate Cox models to avoid overfitting and unstable estimates. The proportional hazards assumption was not formally tested due to the exploratory nature of the analysis and the retrospective data structure. However, the short time-to-failure intervals and limited follow-up duration reduce the likelihood of major time-varying effects.

Patients who were still on treatment at the time of data cut-off or were lost to follow-up were censored at their last known treatment date. The Cox proportional hazards models accounted for right-censored data using standard partial likelihood estimation. No formal sensitivity analyses were conducted for censored observations due to the limited sample size.

Continuous variables were analyzed as continuous measures and, in selected cases, categorized based on clinical cut-offs (example: AST > 25). Categorization was applied to facilitate interpretation and enable analysis using the Cox proportional hazards model. For analytical purposes, the presence or absence of metastases in the liver, lymph nodes, lungs, or bones was treated as a binary variable (yes/no). The IMDC prognostic score was not applied due to missing data for key components (e.g., calcium, neutrophil count, performance status) in a significant proportion of cases.

## 3. Results

The study included 137 patients, consisting of 95 men (69.3%) and 42 women (30.7%), receiving a total of 227 lines of treatment: 137 first-line treatments, 65 second-line treatments, and 25 third- or fourth-line treatments. The longest ongoing treatments were initiated up to 11 years ago (2013), predominantly in patients treated with sunitinib. Of the 227 treatment lines, 47 were ongoing at the time of data cut-off, while 180 were completed due to progression, intolerance, or death. The duration of the completed treatment lines ranged from 14 days (single administration) to 4017 days of treatment.

Table 2 provides the average age of patients receiving different systemic therapies, stratified by treatment line and regimen. The overall average age was slightly lower in second- and later-line settings (59.8 years) compared to first-line treatments (60.8 years), with Everolimus being administered to the youngest subgroup (average 45.3 years) and Axitinib + Avelumab to the oldest (65.9 years).

Sex Distribution by Treatment Line: first-line treatments included 93 male patients out of 137 (67.8%) and second to fourth-line treatments included 48 male patients out of 64 (75.0%). The difference did not reach statistical significance in a Z-test (*p* = 0.27).

Treatment Outcomes (TTF):

Table 3, Table 4, Table 5 and Table 6 present the average, median, and range of time to treatment failure (TTF) for each treatment line and medication evaluated.

Table 3 summarizes time to treatment failure (TTF) outcomes for each first-line regimen used in the cohort. Among first-line therapies, Sunitinib and Pazopanib were associated with the longest median and average TTFs, while Ipilimumab + Nivolumab and Avelumab + Axitinib showed shorter durations. The shortest TTF was observed with Tivozanib and Temsirolimus, which were rarely used.

Table 4 presents time to treatment failure (TTF) data for second-line therapies in patients with mRCC. Nivolumab and Axitinib showed the longest average TTFs among regimens used more frequently, while Cabozantinib was associated with notably shorter treatment durations. Rarely used combinations, such as Nivolumab + Axitinib, showed prolonged TTF in isolated cases.

Table 5 shows time to treatment failure (TTF) for third-line therapies administered in the cohort. Among agents used more than once, Nivolumab had the longest average TTF, while Cabozantinib demonstrated a shorter treatment duration. Everolimus showed moderate performance in two patients, and data for Axitinib were insufficient for interpretation.

Table 6 presents time to treatment failure (TTF) for fourth-line therapies in a limited subset of patients. Nivolumab was the only agent administered in this setting, with a median and average TTF of 611 days (20.3 months), suggesting prolonged benefit in selected patients despite advanced treatment lines.

Predictive Factors Analysis:

Clear cell RCC demonstrated better TTF compared to non-clear cell histologies (RR = 0.66, *p* = 0.0367).

Table 7 shows the results of univariate Cox regression analysis for baseline predictors of time to treatment failure (TTF) in patients receiving Ipilimumab + Nivolumab. Among the variables analyzed, elevated AST (either as a continuous variable or dichotomized at >25 U/L) was significantly associated with shorter TTF (RR = 1.01, 95% CI: 1.0024–1.024, *p* = 0.017; RR = 2.56, 95% CI: 1.15–5.70, *p* = 0.021). Similarly, the combined value of AST + ALT was a significant predictor (*p* = 0.022).

Other variables demonstrated trends toward significance: low hemoglobin (HR = 0.86, *p* = 0.070) and elevated ALT (HR = 1.01, *p* = 0.075) both showed a borderline association with earlier treatment failure.

No statistically significant associations were observed for neutrophils, BMI, calcium, or metastatic burden (including liver, bone, lymph node, or lung involvement). Parameters such as lymphocyte count, creatinine, and total bilirubin were also not predictive of TTF in this subgroup.

Table 8 presents the results of univariate Cox regression analysis for baseline factors associated with time to treatment failure (TTF) in patients treated with Axitinib + Avelumab.

Several variables were significantly associated with shorter TTF in this subgroup. The presence of lymph node metastases (M1 lym) was a strong predictor of early treatment failure (RR = 11.98, 95% CI: 1.657–86.63, *p* = 0.014). Additionally, low lymphocyte count, low creatinine, low body mass index (BMI), and low hemoglobin were all significantly correlated with reduced TTF (*p* between 0.025 and 0.041), suggesting a potential link with immune, nutritional, or functional reserve.

Two variables showed borderline significance—elevated platelet count (*p* = 0.061) and higher neutrophil count (*p* = 0.081)—both suggesting a possible inflammatory component associated with reduced treatment durability. Other parameters, including age, AST, ALT, bilirubin, calcium levels, and presence of metastases at other sites (lung, bone, liver), did not show a statistically significant association with treatment outcomes.

Table 9 presents the results of univariate Cox regression for patients treated with Cabozantinib in second or third line following prior immunotherapy-based regimens.

Several baseline parameters were significantly or borderline associated with shorter time to treatment failure (TTF). Among these, higher platelet count (RR = 1.005, *p* = 0.0223), presence of liver metastases (RR = 2.90, 95% CI: 1.088–7.74, *p* = 0.0332), and elevated ALT (TGP) (RR = 1.025, *p* = 0.0484) were statistically significant predictors of early treatment discontinuation.

Additionally, neutrophil count (*p* = 0.0512), total calcium (*p* = 0.067), serum potassium (*p* = 0.0676), and AST (TGO) (*p* = 0.0768) all showed borderline significance, suggesting a possible role of systemic inflammation, electrolyte imbalance, or hepatic involvement in early Cabozantinib failure.

Other parameters, such as BMI, hemoglobin, creatinine, or metastatic distribution (bone, lung, lymph nodes), did not reach statistical significance. These findings suggest that patients with inflammatory or liver-related baseline abnormalities may experience limited benefit from Cabozantinib in later treatment lines.

Although Nivolumab was administered to a substantial number of patients in the subsequent lines setting, no statistically significant predictive factors for TTF were identified in this group (Table 10). This absence of association may reflect limitations related to sample size and statistical power, or a relatively low variability in baseline clinical and laboratory parameters. Additionally, the heterogeneity of prior therapies and patient selection might have further diluted potential signals, making it challenging to isolate robust predictors in this real-world cohort.

We produced an exploratory Cox model comparing L1 Ipilimumab + Nivolumab vs. Avelumab + Axitinib. While this comparison contrasts different IMDC risk categories, it did not reach statistical significance (RR = 0.842, favoring Ipi + Nivo, but the confidence interval is 0.417–1.69, *p* = 0.63); it nonetheless adds interpretive value regarding treatment duration. Direct comparisons between older TKI regimens (e.g., Sunitinib, Pazopanib) were avoided due to survivorship bias, as many patients in the TKI groups were initiated on therapy in earlier years and represent a preselected population of long-term responders.

**Figure 2:**
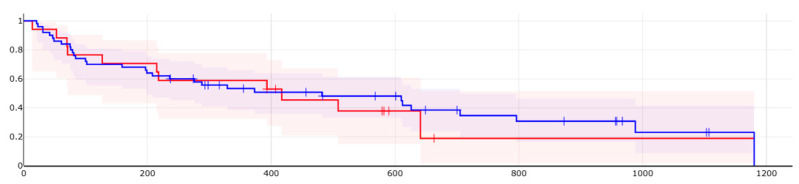
Kaplan–Meier analysis of time to treatment failure (TTF) in patients receiving Ipilimumab + Nivolumab versus Avelumab + Axitinib as first-line therapy. (in blue Ipi + Nivo, in red Ave + Axi, duration in days).

Figure 2 Kaplan–Meier analysis of time to treatment failure (TTF) in patients receiving Ipilimumab + Nivolumab versus Avelumab + Axitinib as first-line therapy.

Treatment allocation was not randomized; Ipilimumab + Nivolumab was preferentially prescribed to patients with intermediate or poor IMDC risk profiles, in accordance with guideline-based clinical practice. The survival curves reflect real-world treatment selection and associated outcomes rather than strictly comparable patient populations.

## 4. Discussion

The findings of this study must be interpreted in light of its retrospective design, non-randomized structure, and the relatively small sample size, all of which may limit the generalizability of the results.

In our analysis, clear cell RCC demonstrated better TTF compared to non-clear cell histologies (RR = 0.66, *p* = 0.0367). This is consistent with a 2017 cohort study of 1943 patients, which reported survival differences primarily between papillary type I RCC and clear cell RCC, but not broadly across all non-clear cell subtypes [33].

Regarding Ipilimumab + Nivolumab, we identified elevated AST (TGO) levels—both as a continuous variable and when exceeding 25 U/L—as statistically significant negative predictive factors for shorter TTF. The combined transaminase score (AST + ALT) further supported this association. Interestingly, BMI did not emerge as a favorable predictor in our analysis, which contrasts with the “obesity paradox” reported in meta-analyses of RCC patients receiving immunotherapy (OS HR = 0.77 for overweight/obese patients) [34]. Another retrospective study of 126 patients with mRCC treated with Nivolumab found a significant positive association between BMI and OS (OS = 40.6 months for BMI ≥ 25 vs. 9.4 months for BMI < 25) [35]. Elevated AST may reflect a more aggressive disease phenotype or occult liver metastases. This observation is supported by prior studies in other cancer types, such as the Gustave Roussy Immune (GRIm) score in early breast cancer, where elevated AST is considered a negative prognostic factor [36].

For Avelumab + Axitinib, the presence of lymph node metastases emerged as a strong negative predictive factor for shorter TTF, while higher absolute lymphocyte counts were associated with longer TTF [37]. A modest association between higher baseline creatinine levels and longer TTF was also observed; although not definitively explained, this finding may relate to differences in muscle mass or nutritional status, which have previously been explored in the context of immune checkpoint inhibitor outcomes [38]. However, this interpretation remains speculative and should be validated in larger studies.

Consistent with the obesity paradox, both higher BMI and higher hemoglobin levels were positively associated with longer TTF in this subgroup. While hemoglobin is already part of the IMDC prognostic model, our findings suggest that it may also have predictive value for TTF in Avelumab + Axitinib treatment.

In the Cabozantinib used in subsequent lines subgroup, negative predictive factors for shorter TTF included elevated ALT, platelet counts, and the presence of liver metastases (RR = 2.904, *p* = 0.0332). These findings align with other real-world evidence identifying poor-risk features such as liver metastases and inflammatory markers as associated with worse outcomes. Notably, previous studies have highlighted higher BMI, prior nephrectomy, and favorable IMDC scores as positive prognostic factors for overall survival in Cabozantinib-treated patients [39], though they have not consistently addressed the impact of liver metastases on TTF specifically.

Higher baseline potassium levels show a tendency for shorter TTF (RR = 7.36, *p* = 0.0676), although this finding is based on a small sample size (*n* = 8) with no abnormal potassium values recorded. This observation warrants further validation.

Although we were unable to demonstrate statistically significant predictive markers for response to nivolumab treatment, in the same second line, a Nivolumab real-world Dutch study (Verhaart et al. 2021) discovered early increases in eosinophil count and neutrophil decreases to be associated with improved TTF (*p* ≤ 0.014), illustrating the value of hematologic dynamics as predictive signals. While our study focused on baseline, easily measurable biomarkers (e.g., AST, lymphocytes, platelets), these findings collectively underscore the need for both baseline and on-treatment markers to optimize early identification of treatment failure in routine practice.

Given the exploratory nature of the study and its real-world design, the sample size was adequate to identify potential predictive signals, though limited cohort sizes for some treatment groups warrant cautious interpretation of the results.

This study has several limitations. First, its retrospective and single-center design may limit the generalizability of the findings. The absence of a control group and the lack of blinding may introduce bias in both treatment selection and outcome interpretation. Additionally, the relatively small sample size in some treatment subgroups reduced statistical power. Time to treatment failure (TTF) was used as a pragmatic endpoint in the real-world setting; however, it may be influenced by non-clinical factors such as physician judgment or patient preference. Despite these limitations, the study provides real-world insights into early treatment failure in mRCC and identifies clinically accessible predictors that may help inform therapeutic decisions and risk stratification in routine oncology practice.

By identifying routine and accessible clinical and laboratory predictors—specific to each regimen—that are associated with shorter time to treatment failure, we aimed to help clinicians optimize treatment sequencing strategies, tailor monitoring intensity, and enhance patient counseling in daily oncology practice.

## 5. Conclusions

In this real-world, single-center, retrospective cohort of patients with non-urothelial metastatic RCC, we observed a progressive loss of patient numbers with each successive treatment line, emphasizing the ongoing clinical challenge of optimizing treatment sequencing for these patients. We identified several negative predictive factors for shorter time to treatment failure (TTF), which differed depending on the treatment received. For patients treated with Ipilimumab plus Nivolumab, elevated AST was associated with shorter TTF. In those receiving Avelumab plus Axitinib, the presence of lymph node metastases, low lymphocyte count, low creatinine, low body mass index, and low hemoglobin was predictive of poorer outcomes. For patients treated with Cabozantinib in subsequent lines, elevated ALT, platelets, and the presence of liver metastases were associated with shorter TTF. No statistically significant predictive factors for TTF were identified in subsequent lines of Nivolumab treatment.

These findings contribute to the growing body of evidence supporting the use of biological and clinical markers to individualize treatment strategies in mRCC. However, the hypothesis-generating nature of these results requires future validation in larger, prospectively designed studies.

## Figures and Tables

**Table 1:** Synthesis of main trials in kidney cancer treatment.

Treatment	Comparator	Year of Publication	HR for PFS (95% CI)	HR for OS (95% CI)	% Grade ≥ 3 Adverse Reactions
Sunitinib, L1 [20]	Interferon alfa	2007	HR = 0.42 (0.32–0.54)	HR = 0.82 (0.66–1.00)	~40%
Pazopanib, L1 [21]	Placebo	2010	HR = 0.46 (0.34–0.62)	HR = 0.91 (0.66–1.25)	~30%
Nivolumab, ≥L2 [22]	Everolimus	2015	HR = 0.88 (0.75–1.03)	HR = 0.73 (0.57–0.93)	~20%
Ipilimumab + Nivolumab, L1 [23]	Sunitinib	2018	HR = 0.82 (0.66–1.02)	HR = 0.63 (0.44–0.89)	~55%
Cabozantinib, L1 [24]	Sunitinib	2016	HR = 0.66 (0.46–0.95)	HR = 0.80 (0.53–1.21)	~60%
Avelumab + Axitinib, L1 [25]	Sunitinib	2019	HR = 0.61 (0.47–0.79)	HR = 0.92 (0.70–1.21)	~50%
Cabozantinib + Nivolumab, L1 [26]	Sunitinib	2021	HR = 0.51 (0.41–0.64)	HR = 0.66 (0.53–0.83)	~55%
Lenvatinib + Pembrolizumab, L1 [27]	Sunitinib	2021	HR = 0.39 (0.28–0.55)	HR = 0.66 (0.49–0.88)	~55%

**Table 2:** Average age of patients by treatment line and regimen.

	Line of Treatment	Average Age (Years)	Number of Patients
Cabozantinib	L1	57.66	3
Ipilimumab + Nivolumab	L1	58.94	50
Pazopanib	L1	60.14	15
All medications L1	L1	60.8	137
Sunitinib	L1	63.87	49
Axitinib + Avelumab	L1	65.94	17
Everolimus	L2–L3–L4	45.3	3
Cabozantinib	L2–L3–L4	58.5	27
All medications L2–L4	L2–L3–L4	59.8	90
Axitinib	L2–L3–L4	60.9	12
Nivolumab	L2–L3–L4	61.7	46

**Table 3:** First-line treatments.

First-Line Treatments	Number of Patients (*n* = 137)	Median TTF Days (Months)	Average TTF Days (Months)	Range Days (Months)
Ipilimumab + Nivolumab	50	178 (5.9)	286 (9.5)	21–1159 (0.7–38.6)
Sunitinib	49	334 (11.1)	616 (20.5)	28–4017 (0.9–133.9)
Avelumab + Axitinib	17	215 (7.2)	248 (8.3)	14–691 (0.5–23.0)
Pazopanib	15	324 (10.8)	508 (16.9)	62–1400 (2.1–46.7)
Cabozantinib	3	242 (8.1)	214 (7.1)	153–248 (5.1–8.3)
Tivozanib	1	30 (1.0)	—	—
Temsirolimus	2	114 (3.8)	231 (7.7)	25-671 (0.83–22.3)

**Table 4:** Second-line treatments.

Line 2 Treatments	Number of Patients (*n* = 65)	Median TTF Days (Months)	Average TTF Days (Months)	Range Days (Months)
Nivolumab	32	277 (9.2)	378 (12.6)	16–1371 (0.5–45.7)
Cabozantinib	19	53 (1.8)	105 (3.5)	20–473 (0.7–15.8)
Axitinib	10	138 (4.6)	392 (13.1)	31–1584 (1.0–52.8)
Everolimus	1	61 (2.0)	—	—
Sunitinib	2	185 (6.2)	185 (6.2)	32–337 (1.1–11.2)
Nivolumab + Axitinib	1	823 (27.4)	—	—

**Table 5:** Third-line treatments.

Line 3 Treatments	Number of Patients (*n* = 23)	Median TTF Days (Months)	Average TTF Days (Months)	Range Days (Months)
Nivolumab	11	122 (4.1)	355 (11.8)	32–1208 (1.1–40.3)
Cabozantinib	8	100 (3.3)	89 (3.0)	35–123 (1.2–4.1)
Axitinib	2	265 (8.8)	265 (8.8)	257–274 (8.5–9.1)
Everolimus	2	122 (4.1)	122 (4.1)	92–153 (3.1–5.1)

**Table 6:** Fourth-line treatments.

Line 4 Treatments	Number of Patients (*n* = 23)	Median TTF Days (Months)	Average TTF Days (Months)	Range Days (Months)
Nivolumab	2	611 (20.3)	611 (20.3)	117–1106 (3.9–36.8)

**Table 7:** Predictive factors analyzed for Ipilimumab + Nivolumab TTF.

Parameter	Average	±SD	*n*	RR	95% Confidence Interval	*p*
AST	24.6	27	45	1.01	1.0024–1.024	0.017
AST > 25	24%		45	2.56	1.15–5.70	0.021
AST + ALT	54.4	61.7	44	1.01	1.0009–1.0115	0.022
Hb	11.79	2.05	45	0.86	0.73–1.01	0.070
ALT	29.6	38.1	44	1.01	0.999–1.015	0.075
Neutrophils	6.24	2.94	45	1.07	0.96–1.18	0.21
Total bilirubin	0.52	0.18	42	4.21	0.37–48	0.25
M1 lym	0.84	0.366	50	0.6413	0.274–1.199	0.31
Leucocytes	8.86	3.07	45	1.06	0.94–1.18	0.34
Body mass index kg/m^2^	26.2	6.8	33	0.96	0.89–1.04	0.35
Calcium (total)	9.43	0.76	39	1.25	0.72–2.15	0.43
M1 oss	0.22	0.414	50	0.707	0.294–1.67	0.43
Age	58.9	12.8	50	0.99	0.96–1.01	0.59
M1 hep	0.28	0.449	50	0.8133	0.348–1.897	0.63
Lymphocytes	1.72	0.65	45	0.89	0.511–1.54	0.67
Creatinine	1.06	0.52	44	1.12	0.55–2.31	0.74
Sex	66% men		50	1.13	0.52–2.42	0.75
M1 pul	0.68	0.4665	50	0.9056	0.433–1.895	0.79
Thrombocytes	338.9	131	45	0.9996	0.997–1.003	0.80

(ALT = alanin amino transpherase, AST = aspartate aminotransferase, Hb = hemoglobin, M1 pul = lung metastases, M1 lym = distant lymph node metastases, M1 hep = liver metastases, M1 oss = bone metastases).

**Table 8:** Predictive factors analyzed for Axitinib + Avelumab TTF.

Parameter	Average	±SD	*n*	RR	95% Confidence Interval	*p*
M1 lym	0.117	0.322	17	11.98	1.657–86.63	0.014
Lymphocytes	1.73	0.96	15	0.356	0.145–0.877	0.025
Creatinine mg/dL	1.14	0.31	15	0.0573	0.004–0.87	0.039
BMI kg/m^2^	29.5	8.3	8	0.819	0.677–0.991	0.040
Hb g/dL	13.3	2.9	15	0.738	0.551–0.997	0.041
Thrombocytes	283.4	97.5	15	1.008	0.999–1.016	0.061
Neutrophils	5.95	2.66	15	1.31	0.967–1.765	0.081
Sex	76% men		17	0.378	0.089–1.610	0.189
M1 pul	0.647	0.4779	17	0.4158	0.130–1.56	0.2098
M1 hep	0.235	0.424	17	0.579	0.122–2.745	0.4919
Total bilirubin mg/dL	0.54	0.15	15	0.261	0.005–14.08	0.509
M1 oss	0.4118	0.4922	17	1.4075	0.403–4.916	0.5922
Leuccocytes	8.42	2.64	15	1.066	0.772–1.472	0.698
ALT UI/L	17.3	10.8	15	0.986	0.918–1.059	0.704
AST UI/L	21.2	10.6	15	0.988	0.923–1.057	0.725
Age	65.9	8.4	17	1.007	0.919–1.103	0.882
Total calcium mg/dL	9.38	0.94	12	1.024	0.32–3.27	0.968

(ALT = alanin amino transpherase, AST = aspartate aminotransferase, Hb = hemoglobin, M1 pul = lung metastases, M1 lym = distant lymph node metastases, M1 hep = liver metastases, M1 oss = bone metastases).

**Table 9:** Predictive factors analyzed for Cabozantinib TTF, only subsequent lines L2–L3 after IO–IO or IO–TKI.

Parameter	Average	±SD	*n*	RR	95% Confidence Interval	*p*
Thrombocytes	318.07	159.53	14	1.005	1.0007–1.009	0.0223
M1 hep	29.60%		27	2.904	1.088–7.74	0.0332
TGP/ALT (U/L)	31.64	27.88	14	1.0249	1.0002–1.05	0.0484
Neutrophils	7.87	6.5	14	1.099	0.999–1.209	0.0512
Calcium total (mg/dL)	9.12	0.71	13	2.675	0.93–7.67	0.067
K+ mmol/L	4.66	0.64	8	7.63	0.863–67.5	0.0676
TGO/AST (U/L)	46.73	65.47	14	1.0086	0.999–1.018	0.0768
BMI kg/m^2^	29.92	7.2	12	0.9313	0.854–1.015	0.1056
Hemoglobin (g/dL)	12.27%	1.61	14	0.804	0.54–1.17	0.261
Leucocytes	12.27	1.6	14	0.8044	0.549–1.177	0.2631
Lymphocytes	1.667	0.779	14	0.595	0.227–1.553	0.2891
M1 oss	77.70%		27	1.972	0.552–7.034	0.2954
Creatinine (mg/dL)	1.1	0.22	14	2.979	0.299–29.61	0.3156
sex	74% males		27	0.73	0.25–2.09	0.56
age (y)	58.5	11.61	27	1.0121	0.939–1.057	0.586
Btotal bilirubin (mg/dL)	76.90%	0.42	13	1.331	0.19–9.17	0.771
ECOG	95.00%	0.75	23	0.917	0.47–1.77	0.799
M1 pul	66.60%		27	1.107	0.39–3.13	0.848
M1 lym	66.60%		27	1.073	0.402–2.86	0.887

(ALT = alanin amino transpherase, AST = aspartate aminotransferase, Hb = hemoglobin, M1 pul = lung metastases, M1 lym = distant lymph node metastases, M1 hep = liver metastases, M1 oss = bone metastases).

**Table 10:** Predictive factors analyzed for Nivolumab used in L2-L3, TTF.

Parameter	Average	±SD	*n*	RR	95% Confidence Interval	*p*
Thrombocytes	268	124	26	1.0032	0.999–1.007	0.116
Calcium total, mg/dL	9.52	0.72	20	0.576	0.258–1.282	0.177
Bilirubin total mg/dL	0.62	0.22	24	4.66	0.459–47.4	0.193
Hb g/dL	12.2	2.8	26	0.878	0.721–1.070	0.199
Sex	75% males		28	1.88	0.630–5.630	0.257
Neutrophils	5.1	4.5	26	1.0414	0.956–1.134	0.352
Age years	62.4	7.4	28	1.025	0.957–1.097	0.481
TGO ASAT U/L	23.4	14.5	25	0.989	0.958–1.020	0.499
Lymphocytes	1.76	0.83	26	1.162	0.721–1.875	0.536
Leucocytes	7.6	4.5	26	1.049	0.967–1.138	0.541
TGP SLAT U/L	17%	8.8	26	0.987	0.931–1.046	0.666
Creatinine mg/dL	1.33	0.77	25	1.013	0.601–1.709	0.959

## Data Availability

The data generated or analyzed during this study are included in this published article or are available from the corresponding author upon reasonable request.

## Abbreviations

The following abbreviations are used in this manuscript:

mRCC	Metastatic renal cell carcinoma
ccRCC	Clear cell renal cell carcinoma
IMDC	International mRCC Database Consortium
ECOG	Eastern Cooperative Oncology Group
BMI	Body Mass Index
AST	Aspartate Aminotransaminase
ALT	Alanine Aminotransaminase
CRP	C-reactive protein
CBC	Complete blood count
TTF	Time to Treatment Failure
PFS	Progression-free survival
OS	Overall survival
M1 oss	Bone metastases
M1 hep	Hepatic metastases
M1 pul	Lung metastases
M1 lym	Lymph node metastases
SD	Standard deviation
N	Number
